# Ten Years of Podoconiosis Research in Ethiopia

**DOI:** 10.1371/journal.pntd.0002301

**Published:** 2013-10-10

**Authors:** Kebede Deribe, Sara Tomczyk, Fasil Tekola-Ayele

**Affiliations:** 1 Brighton and Sussex Medical School, Falmer, Brighton, United Kingdom; 2 Addis Ababa University, School of Public Health, Addis Ababa, Ethiopia; 3 Institute of Tropical Medicine, Antwerp, Belgium; 4 Center for Research on Genomics and Global Health, National Human Genome Research Institute, National Institutes of Health, Bethesda, Maryland, United States of America; Kwame Nkrumah University of Science and Technology (KNUST) School of Medical Sciences, Ghana

## Background

Podoconiosis (endemic non-filarial elephantiasis) is a non-infectious geochemical disease among barefoot subsistence farmers who have long-term contact with irritant red clay soil of volcanic origins. The disease causes progressive bilateral swelling of the lower legs [1], [2]. Previous studies have documented the association between the disease and irritant red clay soils found in areas greater than 1500 metres above sea level, with greater than 1000 mm annual rainfall and average annual temperature of 20°C [1]. The term podoconiosis was coined by Ernest Price, derived from the Greek words *podos* and *konos*, which mean foot and dust, respectively, and imply that the disease is caused by exposure of feet to irritant clay soil [1].

**Figure 1:**
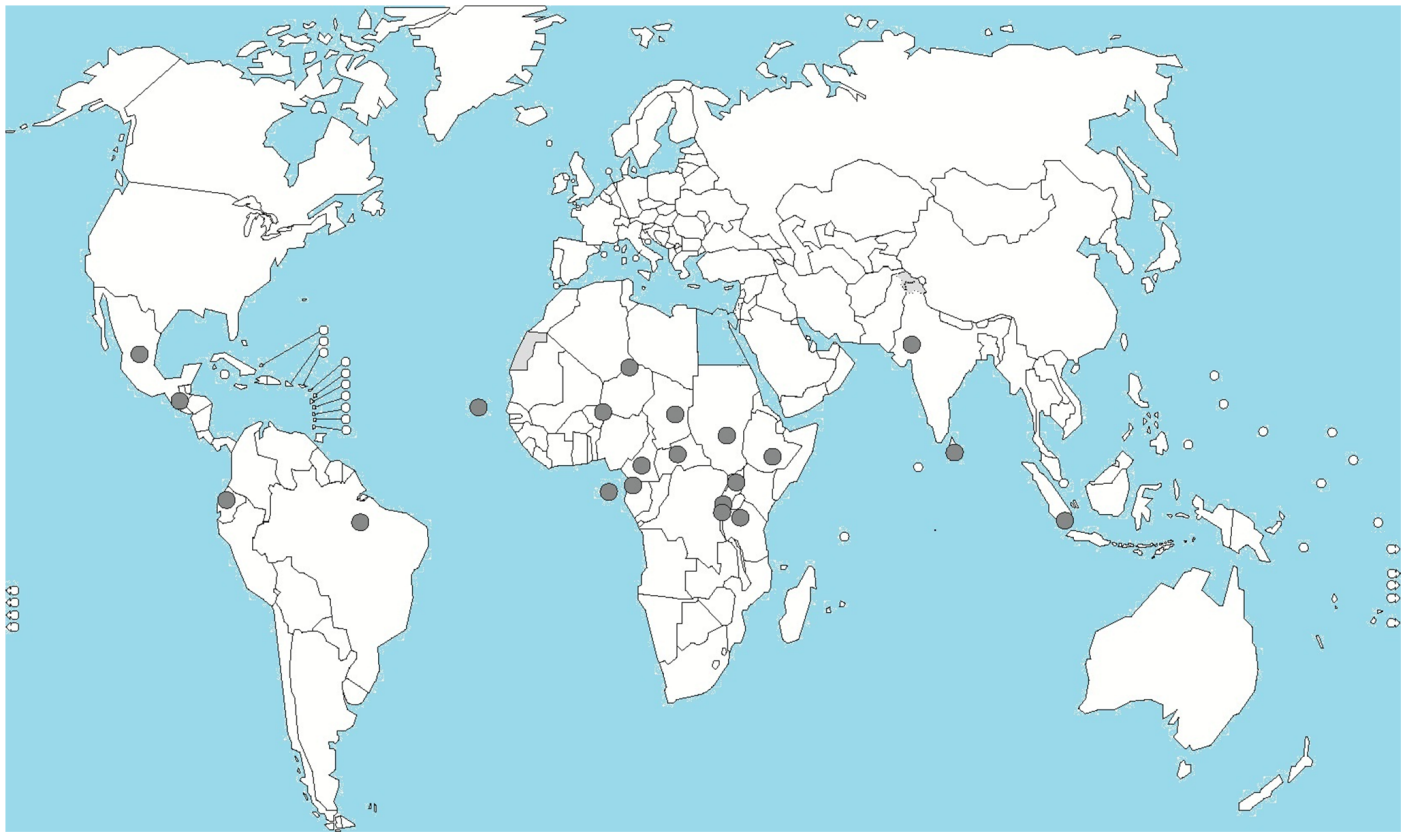
Countries where podoconiosis is endemic or has been described.

Podoconiosis is widely distributed in certain countries on three continents: Africa, South America, and Asia [1]. At least ten African countries have highland areas where the disease is endemic, including Ethiopia, which has the largest number of podoconiosis patients [1], [2]. Podoconiosis is one of the neglected tropical diseases (NTDs) with the greatest potential for elimination; it is preventable if shoes are consistently worn (for example, the disease has disappeared from North African countries, France, Ireland, and Scotland since use of footwear became the norm [1]), and early stages can be successfully treated using a simple lymphoedema regimen (Figure 1).

## The Historical Perspective

Ernest Price, in the early 1970s, conducted extensive research in Ethiopia and in the eastern African region on the aetiology, natural history, distribution, and management of non-filarial elephantiasis [1]. He approached the research through a multi-disciplinary perspective using clinical epidemiology, geology, pathology, and genetics. In his pioneering work, Price identified the possible association between the disease and exposure to red clay soil. This led to the acceptance of podoconiosis as a disease entity distinct from other forms of lymphoedema and to the birth of its present name “podoconiosis.” Much of Price's work was undertaken during a period when involvement of African institutions and investigators in research led by Western investigators was limited, and his remarkable body of research was never linked with interventions. Between 1990, when Price died, and 2000, limited progress was made, and podoconiosis research entered a “Dark Age.”

In 2002, Gail Davey was first introduced to the problem of podoconiosis in one of the southern districts of Ethiopia. Since then, she has led podoconiosis research using a multi-disciplinary approach, including epidemiology, immunology, mineralogy, genetics, bioethics, social sciences, and economics, to describe the disease and its impacts. In addition to Price and Davey approaching podoconiosis from a multi-disciplinary perspective, Davey went the extra mile by partnering with local and international institutions that translated the research into disease prevention and treatment. Several factors have contributed to the success of Davey's program. Unlike Price, Davey has (i) embraced social science and public health research rapidly applicable to education of community members and clinical management, (ii) included service-providing institutions (e.g., the Mossy Foot Treatment and Prevention Association (MFTPA), and International Orthodox Christian Charities, Ethiopia [IOCC]), (iii) engaged a wider range of academic and other institutions in Ethiopia and from around the world (e.g., Addis Ababa University, Armauer Hansen Research Institute [AHRI], funding agencies, and shoe companies), and (iv) most importantly, included local investigators (at the time of writing of this paper, 11 young Ethiopians have completed or are doing their graduate studies on podoconiosis and have been lead authors in 15 peer-reviewed journal publications on podoconiosis on which Davey was also the principal investigator). Contextual factors that have contributed to the success of this program have been increased awareness of the importance of partnership in North–South research collaborations, and the recent global attention focussed on NTDs.

## Major Accomplishments during the Past Ten Years

**Figure 2:**
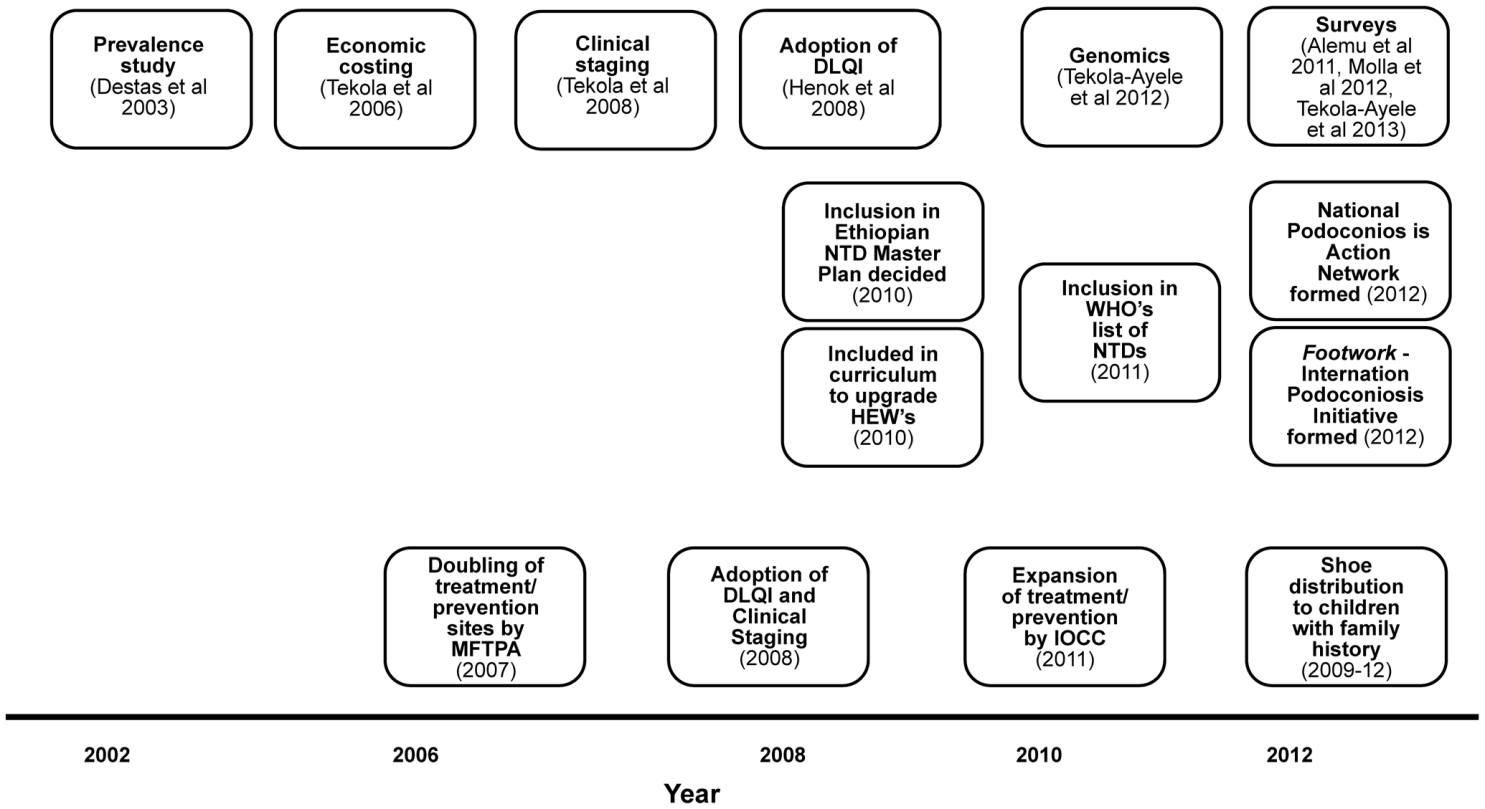
Major achievements resulting from podoconiosis research during the past ten years.

The major accomplishments of the past decade fall into three interlinked categories: scientific research, inclusion of podoconiosis in national and international health agendas, and integration of research findings into clinical management of podoconiosis (Figure 2).

Considering first scientific research, the initial group of podoconiosis studies aimed to describe the burden of the disease in Ethiopia in terms of prevalence, economic cost, and social burden. Studies in different regions of Ethiopia have documented prevalence ranging from 2.8% to 7.4% [3]–[7]. In a zone of 1.5 million people, the cost of podoconiosis in lost productivity was found to exceed US$ 16 million per year [8]. Over half of the patients had impaired physical movement and daily activity, and complications of the disease resulted in considerable morbidity and economic inactivity [3], [7]. Studies have also documented significant social impact of podoconiosis manifested through various forms of stigma against affected individuals [9]. The second important milestone in podoconiosis research was the development of a *de novo* clinical staging system that could be used by community workers with little health training [10] and the adoption of the Dermatology Life Quality Index (DLQI) for measuring quality of life in podoconiosis patients [11]. Third, a ground-breaking genomics study showed that genetic variants near or within the genes *HLA–DQA1*, *–DRB1*, and *–DQB1* confer risk for podoconiosis [12], and a pedigree study on multiply-affected families showed that podoconiosis has a strong genetic component [13].

The aforementioned research milestones contributed significantly to the second major accomplishment—recognition of podoconiosis in national and international health agendas. In December 2010, the Ethiopian Federal Ministry of Health agreed to prioritize podoconiosis control. Subsequently, podoconiosis was included in the National Master Plan for NTDs and in the modules prepared by the Open University for the upgrading of Ethiopian Health Extension Workers. In February 2011, the World Health Organization included podoconiosis in its list of NTDs (http://www.who.int/neglected_diseases/diseases/podoconiosis/en/). In March 2012, Foot*work*, the International Podoconiosis Initiative, was launched, with the vision of eliminating podoconiosis (www.podo.org). In 2012, the civil society organizations (CSOs) that had been working on podoconiosis prevention and treatment in Ethiopia for over 20 years formed the National Podoconiosis Action Network (NaPAN), an umbrella organization that coordinates the work of its member organizations to work towards its vision “to see Ethiopia free of podoconiosis.”

The third major accomplishment has been the translation of research outputs into podoconiosis prevention and treatment. For example, the clinical staging system and DLQI scale have been adopted by CSOs that work on the treatment of podoconiosis to measure treatment outcomes [10], [11]. The genomics research projects informed the MFTPA and encouraged it to tailor prevention efforts towards “high-risk” children [14]. Various podoconiosis prevalence surveys [3]–[7] have also been used to prioritize site selection for the expansion of treatment.

## Challenges Encountered

Challenges in podoconiosis research and control have included (i) total dependence on CSOs for podoconiosis treatment services and limited involvement of government health services, making disease prevention efforts unsustainable, (ii) limited knowledge of mid- and high-level health professionals in Ethiopia about the causes and clinical management of podoconiosis and differential diagnosis from other causes of lymphoedema, (iii) neglect by global health advocates of non-infectious and non-fatal, but socio-economically devastating, diseases of the poor such as podoconiosis, thereby limiting the amount and sources of funding available for research and interventions, and (iv) absence of comprehensive data on the distribution and burden of podoconiosis (i.e., disease mapping) and lack of diagnostic tools, which are necessary for negotiating funding and delivery of treatment and interventions.

## Lessons Learnt

The accomplishments of the past ten years have drawn podoconiosis from the book shelves where it was buried for a ten-year “Dark Age” to some light. Podoconiosis is now a global health topic recognized as such by the World Health Organization (http://www.who.int/neglected_diseases/diseases/podoconiosis/en/), the Wellcome Trust (http://www.wellcome.ac.uk/News/Media-office/Press-releases/2012/WTVM054822.htm), and the US National Institutes of Health (http://www.fic.nih.gov/News/GlobalHealthMatters/Documents/ghmmay-jun2012.pdf). The successes of the journey demonstrate the impact of ten years of community-oriented, intervention-linked, multi-disciplinary research to positively influence the lives of marginalized patients. It also shows that scientific research in a low-income setting and among marginalized communities can impact policy, raise disease profile, and touch hearts across the world. These rewarding accomplishments have also generated research questions that future enquiry may answer with huge dividends for scientific knowledge, disease elimination, and lessons for other diseases.

## Conclusions

The success stories of the past ten years and the recent funding of further research projects such as the mapping of podoconiosis in Ethiopia, an evaluation of podoconiosis lymphoedema management, and a trial of behavioural strategies for promoting footwear imply that elimination of podoconiosis is achievable. Mapping of the disease burden of podoconiosis will help target resources, monitor control progress, and advocate for investment in podoconiosis prevention, control, and ultimately elimination. The studies evaluating lymphoedema management and behavioural interventions are a prerequisite for the scale-up of these interventions. Capitalizing on filarial lymphoedema management experience and research outcomes will help update the treatment options for podoconiosis [15]. After defining the target population and identifying the best approaches through research and in consultation with international experts, the goal of elimination can be fulfilled “within our lifetimes” as stated in the ambitious vision of Foot*work*, the International Podoconiosis Initiative.
